# SSCS: A Stage Supervised Subtyping System for Colorectal Cancer

**DOI:** 10.3390/biomedicines9121815

**Published:** 2021-12-02

**Authors:** Lan Zhao, Yi Pan

**Affiliations:** 1 Department of Medicine, Stanford University, Palo Alto, CA 94305, USA; 2 Shenzhen Institute of Advanced Technology, Chinese Academy of Sciences, Shenzhen University Town, Shenzhen 518055, China; yi.pan@siat.ac.cn

**Keywords:** colorectal cancer, tumor heterogeneity, stage supervised CRC subtypes (SSCS), tumor-infiltrating cell

## Abstract

Colorectal cancer (CRC) is heterogeneous and deadly, and the exact cause of the disease is unknown. Recent progress indicated that CRC is not a single disease, but a group of diseases with significant heterogeneity. Three previous CRC subtyping systems: microsatellite instability (MSI), consensus molecular subtypes (CMS), and tumor-node-metastases (TNM) stage were evaluated for their molecular and clinical implications. Results suggested that the MSI and CMS systems are prognostic and predictive mostly in early-stage CRC. As the stage remains an influential factor for CRC subtype analysis, we developed a new subtyping system named stage supervised CRC subtypes (SSCS), in order to better stratify CRC biologically and clinically. Our subtyping system can be used to classify CRC patients into five subtypes (SSCS1-5). SSCS1 was found to have the highest frequency of MSI-H cases compared to the remaining four subtypes. SSCS2 had the most favorable prognosis, whereas the worst prognosis was seen in SSCS4. SSCS3 had cell cycle and metabolism-related gene sets upregulation, and SSCS5 subtype was enriched with amplicon-associated gene sets. Moreover, tumor-infiltrating fibroblast was found to be predictive for poor disease-free survival (DFS) only within the SSCS4 subtype. Conventional dendritic cells (cDC), on the contrary, were associated with favorable DFS in the SSCS3 subtype. Our study provides a new subtyping system SSCS, which can be used for better stratify CRC patients compared to current standards. Further exploration of the subtype-specific cell types has the potential to be novel therapies for CRC.

## 1. Introduction

Colorectal cancer (CRC) starts in the colon or rectum, and is the third most prevalent cancer in the world. CRC is more common in developed countries than in developing countries. Genetic and environmental risk factors such as family history, aging, smoking, obesity, physical inactivity, and chronic inflammation are strongly linked to CRC.

CRC is heterogeneous in terms of distinct clinical outcomes and diverse molecular features. Thus, clinical stage and molecular status have been extensively used to classify CRC into homogeneous groups. Tumor-node-metastases (TNM) staging system as recommended by the American Joint Committee on Cancer (AJCC) is to determine the extent of the cancer or how far it has spread. CRC can be classified into four major stages (I-IV) based on the TNM system. The clinical outcomes of CRC vary greatly by the stage at the time of diagnosis [1,2]. Those diagnosed with early stages of CRC (stages I and II) generally have higher survival rate than advanced stages cases (stages III and IV) [1]. Two distinct genome instabilities, namely chromosomal and microsatellite instability (CIN and MSI) are hallmarks of CRC, representing useful systems to stratify patients for treatment. Aneuploidy can lead to CIN, and about 70% CRC cases have CIN, where chromosomes are incorrectly duplicated or deleted [3]. Loss of heterozygosity (LOH), mitotic recombination and gene conversion contribute to the CIN phenotype. However, markers and criteria for CIN have not been clearly confirmed. In addition, LOH analyses are prone to many inaccurate results [4]. Defect in DNA mismatch repair (dMMR) can lead to MSI [5]. Microsatellites, also called simple sequence repeats (SSRs), which are widely distributed throughout the genomes of many organisms. The majority of microsatellites are located in the non-coding regions and are without biological effect [6]. Others are presented in coding or regulatory regions, which can affect phenotypes and predisposition to diseases. MSI is typically detected by PCR-based amplification of specific microsatellite markers [7]. A small portion (~15%) of CRC arises from the MSI [5]. High frequency microsatellite instability (MSI-H) CRCs are heavily infiltrated by immune cells, making them ideal candidates for immunotherapy, and are associated with favorable outcomes [8,9]. Although MSI status can guide immunotherapy treatment decisions, its prognostic and predictive relevance is often limited [10,11].

With the advance of high-throughput molecular technologies, a growing number of large-scale genome-wide profiling studies were conducted to investigate the CRC heterogeneity in greater depth than before [12,13,14,15,16,17]. The most recently well recognized subtyping system for CRC developed by the Colorectal Cancer Subtyping Consortium (CRCSC) suggested that there are four consensus (CMS1-CMS4) and one non-consensus (NOLBL) subtypes of CRC [18]. CMS1 (14%) is microsatellite unstable, hypermutated, and have strong immune activation; CMS2 (37%) has marked WNT and MYC signaling activation; CMS3 (13%) tumors displayed prominent metabolic dysregulation; CMS4 (23%) malignancies have noticeable transforming growth factor–β activation, and stromal invasion; and the remaining 13% are NOLBL samples without assigned consensus labels CMS subtypes have been shown to provide molecularly and clinically distinct clusters compared to CIN and MSI groups. However, as the majority of the patients included in the original identification of CMS subtypes derived from early stage CRC, making it difficult to provide prognostic values for advanced cases [19,20,21].

Thus, in our study, we first systematically evaluated the clinical relevance and molecular features of previous CRC subtyping systems; followed by developing a new CRC subtyping system SSCS, taking the tumor stage as an independent factor; and lastly, characterization of the identified subtypes.

## 2. Methods

### 2.1. CRC Cohort Datasets

The datasets used in the study include: TCGA colorectal dataset (TCGA in short), CPTAC colon cancer dataset (CPTAC in short), and four CRC Gene Expression Omnibus microarray datasets (GSE14333, GSE39582, GSE33113, and GSE37892; GEO in short) (Table 1).

TCGA mRNA expression profile was downloaded from the TCGAcrcmRNA package (version 1.14.0), which contains 450 primary CRC level 3 (processed) data generated by HiSeq and GenomeAnalyzer platforms. Processed CPATAC mRNA expression data, which contains 105 primary tumors, was downloaded from LinkedOmics (http://linkedomics.org/cptac-colon/; Accessed on 15 August 2019). Patient clinical metadata such as age, gender, MSI status, CMS labels, and patient survival information were downloaded from cBioPortal (http://www.cbioportal.org; Accessed on 15 August 2019), LinkedOmics and/or associated publications [18,22]. Processed and merged GEO mRNA expression and clinical data of 914 CRC (only non-MSI-H patients were included) were obtained from the mcsurvdata package (version 1.11.0) [23]. The details on processing of raw Affymetrix microarray samples, correcting potential technical effects (such as sample’s center of origin, batch, age, gender, TNM stage and MSI status), and merging the four microarray datasets were described in the associated vignettes [23].

### 2.2. Gene Set Enrichment Analysis (GSEA)

Before GSEA, limma package (version 3.48.3) [24] was used for differential expression analysis between one patient group versus the remaining groups in the transcriptome data. The resulting fold changes were used as the inputs for GSEA.

MsigDB annotated gene set collections (version 7.2 C2 and C5) with the number of genes ranging from 10 to 500 were selected for GSEA analysis implemented in the piano package (version 2.10.0) [25]. The *p*-values were estimated by GSEA with 1000 permutations and adjusted by calculating the false discovery rates (FDR). The other parameters were set as default. Top significantly dysregulated gene sets (FDR adjusted *p*-value < 0.05) ranked by enrichment scores were chosen for each patient group, respectively, and used for heatmap visualization. Specifically, a data matrix was generated with rows defined by the selected enriched/depleted gene sets, and columns by −log10 (adjusted *p*-value) for each patient group. Hierarchical clustering was then used to cluster the gene sets into groups. Finally, a heatmap was plotted using the package pheatmap (version 1.0.12) [26] to visualize the gene sets patterns among patient groups.

### 2.3. Survival and Cox PH Regression Analysis

Kaplan–Meier method and log-rank test were used to estimate and compare disease free survival (DFS) and overall survival (OS) among patient groups. Kaplan–Meier survival analysis with pairwise comparisons between patient groups were also performed by using the survminer R package (version 0.3.0) [27]. FDR corrected *p*-values of less than 0.05 were considered statistically significant.

Multivariate Cox PH regression model was used to estimate hazard ratios (HRs) with 95% confidence intervals (CIs) for DFS and OS among patient groups. Covariates in the model included age at diagnosis, gender, CIN status (high and low; when available), and tumor site (left and right). CRC originating in the descending colon, sigmoid colon, rectum, splenic flexure, or rectosigmoid junction were considered left-sided cancers; and CRC originating in the ascending colon, cecum, hepatic flexure, or transverse colon were categorized as right-sided tumors [28]. Ggforest function from the survminer package (version 0.3.0) [27] was used to construct forest plots, which showed HRs and log-rank *p*-value for DFS and OS among patient groups.

### 2.4. Unsupervised Clustering and Random Forest Classifiers

TCGA early and advanced CRC cases were employed for unsupervised classification by cluster analysis, respectively. DFS-associated genes were pre-selected by univariate Cox PH regression analysis, with Wald test *p*-values no more than 0.05. Highly variable genes with MAD scores greater than 0.5 were then chosen for consensus clustering using the ConsensusClusterPlus package (version 4.1.0) [29]. Consensus clustering consisted of 1000 iterations of PAM clustering for each rank k (from 2 to 8), with 0.9 subsampling ratio, and agglomerative average linkage and Spearman correlation was conducted to identify the attributes of each consensus cluster. Gap statistics were calculated over a range of clusters k (from 2 to 8) to determine the optimal cluster number.

The top 50 most important features were selected to construct random forest classifiers for CRC early- and advanced-stage cases in the TCGA cohort, respectively. The classifiers were built with 1000 trees using the randomForest package (version 4.6-14) [30] in R. The two classifiers were then employed to assign patients in the CPTAC and GEO cohorts into corresponding subtypes.

### 2.5. Statistical Analyses

Statistical analyses were conducted by the software R, version 3.6.3. The enrichment scores of infiltrated-immune and stroma cells for each sample was estimated using xCell (xcell.ucsf.edu; Accessed September-2019) [31]. Cox PH analysis was performed for each cell type to identify independent predictors of DFS and OS, respectively. The statistically significant cell types in the univariate Cox analysis (Wald test *p*-value less than 0.05) were selected for further investigation. Pairwise Wilcoxon rank-sum test was performed to evaluate the differences of continuous variables (such as age, ImmuneScore, and StromaScore) among patient groups. Chi-squared test was used to investigate the associations of CRC subtypes with gender, MSI, and CMS labels. FDR adjusted *p*-value of less than 0.05 was considered statistically significant for all tests.

## 3. Results

### 3.1. Evaluations of Three Previous CRC Subtyping Systems

Three CRC subtyping systems (TNM stage, CMS and MSI status) were investigated in terms of their biological implications and clinical relevance. CRC can be grouped into four major stages (I-IV) based on the TNM system. Stage II and stage III CRC make up most of all cases in the TCGA, CPTAC, and GEO datasets (Figure S1). As the tumor progresses from the curable non-invasive stage (I-II, early) to the metastatic stage (III-IV, advanced) with a less favorable prognosis, we consider the tumor stage as a binary variable (early and advanced) in the analysis. Based on mRNA expression profiles, CRC was classified into four consensus subtypes (CMS 1-4) with distinguishing molecular and clinical features and one non-consensus subtype (NOLBL) [18]. According to MSI status, about 15% of CRC were MSI-H, where the microsatellite markers were unstable, in contrast to the remaining 85% non-MSI-H cases.

**Figure 1:**
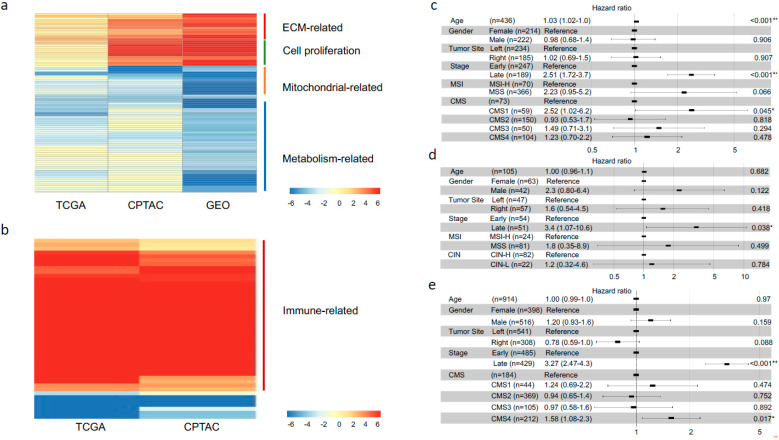
Molecular and clinical relevance of three previous CRC subtyping systems. (**a**). A *p*-value heatmap representing dysregulated gene sets between advanced- and early-stage of CRC (early-stage was used as control). (**b**). A *p*-value heatmap representing dysregulated gene sets between MSI-H and non-MSI-H CRC (non-MSI-H group was used as control). In the heatmaps, columns correspond to different cohorts, and rows to gene sets. Values in the heatmaps were −log10 (FDR adjusted *p*-value) transformed. Red color indicates up-regulated gene sets, and green means down-regulated gene sets in the control groups. Gene sets were grouped into different functional categories, with representative functional names shown on the right hand of the heatmaps, respectively. Forest plots of Cox PH regression models illustrated the HRs, 95% CIs and log-rank *p*-values for different subtyping systems (stage, MSI, CMS, etc.) and confounder factors (age, gender, etc.) for DFS in the TCGA (**c**), CPTAC (**d**), and GEO (**e**) cohorts. Log-rank *p*-value significance levels were given by stars: * <0.05, and ** <0.01.

A total of 436 CRC patients from the TCGA dataset have clinical stage information, and among them there were 247 early- and 189 advanced-stage cases, respectively (Table 1). CPTAC dataset contains 54 (early) and 51 (advanced) colon cancer patients. A total of 914 CRC patients from four independent GEO datasets were combined into one dataset, namely GEO. Of them, 485 were from early stages, and 429 were from advanced stages (Table 1). Gene-level fold changes between advanced- and early-stage cases for these three datasets’ transcription data were used as the inputs for Gene set enrichment analysis (GSEA), respectively. A total of 92 most highly dysregulated common pathways among the three datasets (FDR adjusted *p*-value < 0.001; Table S1) were selected for heatmap visualization. Consistent results were observed among the three cohorts such as gene sets were regulated towards the same direction (both up- and down-regulation), and more gene sets were down-regulated than up-regulated in advanced cases (65 vs. 27) (Figure 1a). Down-regulated gene sets include multiple mitochondrial- and metabolism-related signing pathways, however, extracellular matrix (ECM) and cell proliferation-associated gene sets were up-regulated in advanced-stage CRC (Figure 1a). Taken together, this may imply that as tumor progression proceeds, CRC epithelial cells decreased their metabolism and, on the contrary, showed rapid proliferation and metastasis.

CMS and MSI status labels were obtained from the associated data sources [18,22,23]. The associations between CMS labels and patients’ molecular features were well described in [18]. There were 70 (16.0%) and 24 (22.8%) MSI-H patients in the TCGA and CPTAC datasets, respectively (Table 1). No MSI-H patients were present in the combined GEO dataset, thus was excluded for the GSEA analysis regarding the MSI status. A total of 537 common gene sets were significantly dysregulated between MSI-H and non-MSI-H patients in the TCGA and CPTAC datasets (FDR adjusted *p*-value < 0.001; Table S2). More than 99% of these gene sets were up-regulated in the MSI-H patients (Table S2 and Figure 1b), these include numerous innate and adaptive immune pathways, which agree well with previous studies [8,9].

We next used the Cox proportional hazard (PH) regression model to evaluate the effects of the three subtyping systems on patients’ disease-free survival (DFS) and overall survival (OS), respectively. Confounder factors such as age, gender, tumor site (left and right), and CIN status (H: high and L: low) were also evaluated when available. Age hazard ratio (HR) values were all close to 1, indicating that age did not affect survival (Figure 1c–e and Figure S2). Gender, tumor site, MSI status, and CIN (when available) all showed no significant effects (log-rank *p*-value > 0.05) on either DFS or OS (Figure 1c–e and Figure S2). Advanced-stage was a significant poor prognostic factor for DFS (log-rank *p*-value < 0.05; HR >1), and this was also true for the CPTAC cohort (Figure 1d), where relatively shorter follow up time (<15 months) and fewer patients (~100) were available compared to the other two cohorts (Figure 1c,e). Advanced-stage was also a poor prognostic factor for OS in the TCGA cohort (Figure S2). CMS labels were not evaluated in the CPTAC dataset, as too short survival time to do sufficient estimation (Figure 1d). CMS1 was a poor prognostic factor for DFS in the TCGA dataset, and the HR was 2.52 (95% confidence interval: 1.02–6.2), whereas CMS4 predicts poor DFS in the GEO dataset with HR as 1.58 (1.08–2.3) (Figure 1c,e). CMS labels showed inconsistent effects on survival (specifically DFS), implying that further investigations are necessary.

### 3.2. TNM Stage Remains an Influential Factor for CRC Subtype Analysis

We then evaluated the combination effects of the three previous CRC subtyping systems on clinical significance and biological implications. There will be four different scenario options to consider: Stage + MSI, Stage + CMS, MSI + CMS, and Stage + MSI + CMS. The last scenario was not considered due to lack of data and complexity in the subsequent analysis. As the MSI classification results were more skewed (15% MSI-H vs. 85% non-MSI-H) compared to the Stage (~51% early vs. 49% advanced) and MSI systems (14% CMS1 vs. 37% CMS2 vs. 13% CMS3 vs. 23% CMS4); and the combination of MSI + CMS systems will produce 2 × 4 = 8 clusters, which will further decrease its statistical significance, the scenario of MSI + CMS was also not considered in the study.

**Figure 2:**
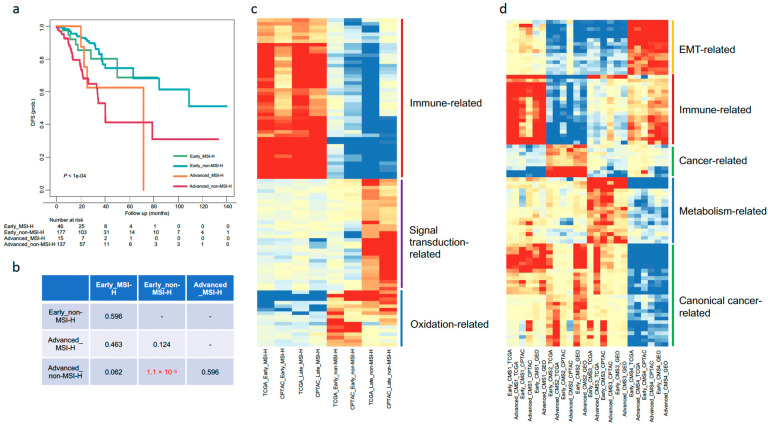
Stage_MSI and Stage_CMS molecular and clinical implications. (**a**). Kaplan–Meier survival curve comparing DFS of the four groups (Early_MSI-H, Early_non-MSI-H, Advanced_MSI-H, and Advanced_non-MSI-H) in the TCGA cohort. The indicated *p*-value was calculated with the log-rank test. (**b**). Summary table of the pairwise comparisons of survival curves between the four groups. Survival difference was tested using the log-rank test, with FDR adjusted *p*-values less than 0.05 were considered statistically significant and displayed in red color. (**c**). A *p*-value heatmap representing dysregulated gene sets between early_MSI-H, early_non-MSI-H, advanced_MSI-H, and advanced_non-MSI-H groups. (**d**). A *p*-value heatmap representing dysregulated gene sets between CMS 1-4 (in early stage) and CMS 1-4 (in advanced-stage) groups. In the heatmaps, columns correspond to different groups, and rows to gene sets. Values in the heatmaps were −log10 (FDR adjusted *p*-value) transformed. Red color indicates up-regulated gene sets, and green means down-regulated gene sets in the corresponding groups. Gene sets were grouped into different functional categories, with representative functional names shown on the right hand of the heatmaps accompanied by a colored line, respectively (such as maroon for immune-related, orange for EMT-related, and blue for metabolism-related gene sets).

For the Stage + MSI scenario (stratifying the patients both by Stage and MSI systems), patients were separated into four groups: Early_MSI-H, Early_non-MSI-H, Advanced_MSI-H, and Advanced_non-MSI-H (Table 1). Groups were compared to each other in the TCGA and CPTAC cohorts. MSI-H patients were more likely to be in early-stage than advanced-stage in both TCGA (53 in early vs. 17 in advanced) and CPTAC (14 vs. 10) datasets (Table 1). In the TCGA cohort, where the patient numbers were sufficient to do pairwise comparisons of survival curves between groups, indicated that the Advanced_non-MSI-H patients have poorer DFS and OS compared to Early_non-MSI-H patients (FDR adjusted *p* < 0.05) (Figure 2a,b and Figure S3). All the other paired differences were not significant (Figure 2b and Figure S3). Fold changes between one group and the others were employed for GSEA analysis. A total of 94 most highly dysregulated common pathways between TCGA and CPTAC datasets (FDR adjusted *p*-value < 0.001; Table S3) were selected for heatmap visualization. We found multiple immune-related pathways were up-regulated in the four MSI-H patient groups (TCGA_Early MSI-H, TCGA_Advanced-MSI-H, CPTAC_Early MSI-H, and CPTAC_Advanced-MSI-H) (Figure 2c). For non-MSI-H groups, several signal transduction-related gene sets were enriched in the advanced cases, and few oxidation-associated pathways were highly enriched in early cases (Figure 2c). In sum, we saw significant clinical and molecular differences between early and advanced cases in non-MSI-H patients under this scenario. However, for MSI-H patients, we did not see significant differences between early and advanced cases.

363 (83.2%) out of the 436 patients in TCGA dataset have available CMS labels (excluding those with NOLBL) were selected for the Stage + CMS scenario investigation. Similarly, 84 (80.0%) and 730 (79.9%) patients in CPTAC and GEO datasets were chosen under this scenario. Thus, a total of eight groups generated for each dataset: CMS1-4 (in early-stage cases) and CMS1-4 (in advanced-stage cases) (Table 1). Fold changes between one group versus the others in a dataset were employed for the GSEA analysis. To better compare the overall gene sets patterns, 89 most highly dysregulated common pathways across the three datasets (FDR adjusted *p*-value < 0.001; Table S4) were selected for heatmap visualization. The enriched gene sets within each CMS clusters agree well with previous report [18], such as immune-related pathways enriched in CMS1; a large collection of canonical cancer-associated pathways up-regulated in CMS2 and CMS1; metabolic pathways in CMS3; and EMT-related pathways enriched in CMS4 (Figure 2d). However, CMS labels showed inconsistent effects on DFS as shown before, that is CMS1 was a poor prognostic factor in the TCGA dataset, whereas CMS4 predicts poor DFS in the GEO dataset (Figure 1c,e). The situation was even worse for OS, where no significant differences were observed among the CMS labels (Figure S2). Note that we used the NOLBL (unlabeled samples) as the reference group, while Guinney et al. [18] chose the CMS2 as the reference for the multivariate Cox analysis. As the DFS seems to be a better clinical endpoint here, we only consider the DFS under this scenario. Although not reaching statistically significant differences when performing the pairwise comparisons of survival curves in the TCGA early and advanced cases, respectively, CMS1 was more likely to be a poor prognostic factor for DFS in the early patients than in advanced patients (Figure S3). More importantly, from the GEO dataset, pairwise comparisons of survival curves between CMS subtypes, indicated that CMS4 predicts poor DFS in the early cases (CMS4 vs. CMS2, CMS4 vs. CMS 3; BH adjusted *p* < 0.05), but not in the advanced cases (Figure S3).

Taken together, we evaluated the clinical significance and biological implications of the Stage + MSI and Stage + CMS scenarios, and found that these two scenarios work well only for a subset of the patients, that is, Stage + MSI for non-MSI-H cases and Stage + CMS for early patients. Altogether, the TNM stage remains an influential factor for CRC subtype analysis. This motivates us to develop a new system to better stratify CRC in terms of prognostic and oncologic values, taking the TNM stage as an independent factor.

### 3.3. Identification of Five Stage Supervised CRC Subtypes

In our subtyping system, namely stage supervised CRC subtypes (SSCS), we began by employing a supervised binary splitting of CRC patients into two large groups (early and advanced); followed by applying an unsupervised clustering of these two groups, separately; early and advanced classifiers were then built to assign patients into corresponding subtypes. TCGA data was used as the training set, CPTAC and GEO were considered for the two validation datasets (Table 1).

**Figure 3:**
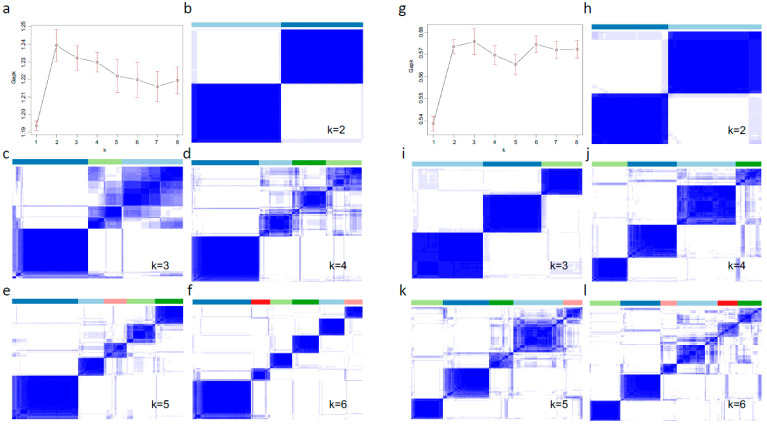
Identification of five subtypes for CRC. The Gap statistics distribution plots with corresponding rank k (from 2 to 8) in the early-stage (**a**), and advanced-stage cases (**g**). The consensus matrices resulting from the consensus PAM clustering were displayed as heatmaps for each rank k (from 2 to 6) that contain the cluster memberships for early-stage (**b**–**f**), and advanced-stage cases (**h**–**l**).

A total of 247 early and 189 advanced cases can be found from the TCGA cohort as mentioned above (Table 1). First, 1850 genes were selected with Wald test *p*-value no more than 0.05 under univariate Cox PH model analysis in the early CRC cases. Then, 1111 genes with MAD > 0.05 were subsequently chosen for consensus clustering. The number of genes selected for advanced cases were 1079 for pre-selection, and 533 for final unsupervised learning, respectively (Table S5). The consensus matrices resulting from the consensus PAM clustering were displayed as heatmaps for each rank k (from 2 to 6) that contains the cluster memberships (Figure 3b–f,h–l). Gap statistics were calculated to determine the optimal cluster numbers, and a peak was found at k = 2 for early cases, and k = 3 for advanced cases (Figure 3a,g). Thus, a total of 5 subtypes (SSCS1-2 for early cases, and SSCS3-5 for advanced cases) were determined in the TCGA cohort. The distribution of our subtyping system (SSCS) in the TCGA cohort was SSCS1 29.6%, SSCS2 27.1%, SSCS3 18.1%, SSCS4 10.3%, and SSCS4 14.9% (Table 1).

In order to classify CRC patients, we built two random forest classifiers (one for early cases and the other for advanced cases) based on the top 50 genes selected according to the feature importance ranking scores in the TCGA early and advanced cases, respectively (Table S5). Classifiers’ misclassification rates in the training dataset (TCGA) were all zeros. 54 and 485 early cases as well as 51 and 429 advanced cases can be found from the CPTAC and GEO cohorts, respectively. The classifiers were then employed to classify the patients from the CPTAC and GEO cohorts into corresponding subtypes: SSCS1-5 (Table 1). SSCS1 was the largest subtype, accounting for approximately 27.6–29.6% of all cases in each cohort, followed by SSCS2 (~25%). The smallest subtype was SSCS4, with the percentage of patients less than 10.5% in the three cohorts, respectively (Table 1).

### 3.4. Molecular Features of the Five Subtypes

**Figure 4:**
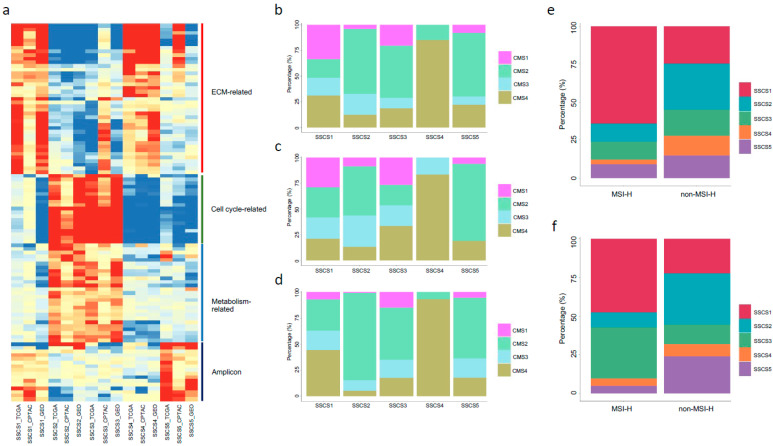
SSCS subtypes molecular features and comparisons with other subtypes. (**a**). A *p*-value heatmap representing dysregulated gene sets between the five SSCS subtypes. In the heatmap, columns correspond to different subtypes in the three cohorts, and rows to gene sets. Values in the heatmap were −log10 (FDR adjusted *p*-value) transformed. Red color indicates up-regulated gene sets, and green means down-regulated gene sets in the corresponding groups. Gene sets were grouped into different functional categories, with representative functional names shown on the right hand of the heatmap accompanied by a colored line, respectively (such as light red for EMT-related, light green for cell cycle-related, and blue for metabolism-related gene sets). The patient’s proportion bar plots illustrate the percentage intersections between the SSCS and CMS labels in the TCGA (**b**), CPTAC (**c**), and GEO (**d**) cohorts. The patient’s proportion bar plots illustrate the percentage intersections between the SSCS and MSI labels in the TCGA (**e**) and CPTAC (**f**) cohorts.

Fold changes between a subtype versus the remaining subtypes in a cohort were employed for GSEA analysis, respectively. A total of 103 most highly dysregulated gene sets selected from the TCGA cohort (FDR adjusted *p*-value < 0.05; Table S6) were used for heatmap visualization. In the heatmap, gene sets with the absolute values of −log10 (adjusted *p*-value) greater than 1.3 (adjusted *p*-value < 0.05) were considered significantly altered. Gene sets were basically regulated towards the same direction (both up- and down-regulation) among the three cohorts (TCGA, CPTAC, and GEO), although some were not statistically significant in the CPTAC cohort (Figure 4a). SSCS1 and SSCS4 subtypes were enriched with ECM-related gene sets; SSCS2 and SSCS3 subtypes have cell cycle- and metabolism-related gene sets upregulation; and SSCS5 subtype enriched with amplicon associated gene sets (Figure 4a and Table S6). Moreover, subtype-specific enriched/depleted patterns can be found from the gene sets heatmap. For example, immune-related gene sets were up-regulated in SSCS1, and ECM related gene sets were down-regulated in SSCS2 (Figure 4a and Table S6). Through functional characterization of the SSCS subtypes illustrates the underlying mechanisms and pathophysiology of CRC.

We next investigated the relationships among SSCS labels with patients’ characteristics, and the results suggested no significant associations between SSCS with patients’ age and gender (data not shown). Patients’ subtyping results by using CMS and MSI systems were also compared to the SSCS labels. From the TCGA cohort, 85% of the patients with the SSCS4 labels were CMS4 patients, and the remaining 15% were from CMS2; in contrast to the SSCS5, where 62% of the patients were with the CMS2 labels (Figure 4b). Similar trends can be seen from the CPTAC and GEO cohorts, such as SSCS4 patients corresponding to CMS4, and the large proportion of the patients from SSCS2 and SSCS5 have CMS2 labels (Figure 4c,d), although the patient’s proportions have minor changes compared to the TCGA cohort (Figure 4b). The frequency of MSI-H cases was highest in SSCS1 (64.2% in TCGA, and 47.6% in CPTAC) compared to the remaining four subtypes (Figure 4e,f).

### 3.5. Prognostic Value of SSCS

**Figure 5:**
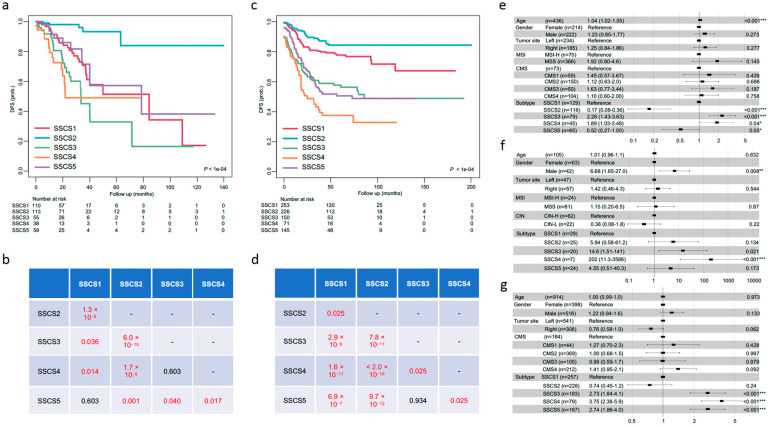
SSCS subtypes clinical characteristics. Kaplan–Meier survival curves comparing DFS of the five SSCS subtypes in the TCGA (**a**) and GEO (**c**) cohorts. The indicated *p*-values were calculated with the log-rank tests. Summary tables of the pairwise comparisons of survival curves between the five SSCS subtypes in the TCGA (**b**) and GEO (**d**) cohorts. Survival differences were tested using the log-rank tests, with FDR adjusted *p*-values less than 0.05 were considered statistically significant and displayed in red colors. Forest plots of Cox PH regression models illustrated the HRs, 95% CIs and log-rank *p*-values for different subtyping systems (SSCS, MSI, CMS, etc.) and confounder factors (age, gender, etc.) for DFS in the TCGA (**e**), CPTAC (**f**), and GEO (**g**) cohorts. Log-rank *p*-value significance levels were given by stars: * <0.05, ** <0.01, and *** <0.001.

There were significant differences in both DFS and OS among the five SSCS subtypes (Figure 5a–d and Figure S4). Pairwise comparison of survival differences among patient groups showed that SSCS2 had the best DFS and OS; SSCS4 had the worst; and the remaining three subtypes had intermediate survivals (Figure 5b,d and Figure S4). Multivariate Cox PH regression analysis supported these findings (Figure 5e–g). The associations of SSCS labels with patient survival adjusted for age, gender, and other factors were significant (log-rank *p*-value < 1 × 10^−4^) with high HRs for SSCS4 and SSCS3, and low HRs for SSCS2, implying that SSCS4 is a poor prognostic factor for DFS, which has been observed from the three cohorts; and SSCS2 had significantly improved DFS in the TCGA cohort (HR 0.17, 95% CI 0.08–0.36, *p* < 0.001), although the difference in the GEO and CPTAC cohorts were not statistically significant (Figure 5e–g).

The enrichment scores for 64 cell components in the tumor microenvironment (TME), spanning multiple innate and adaptive immune, extracellular matrix, and other cell types were estimated by using a gene signature-based method, xcell [31]. Three additional scores (immune, stroma, and microenvironment scores) were obtained by summing all immune, stromal, and immune plus stromal cell types, respectively [31], which were used to represent the tumor purity of each sample. For the TCGA training cohort, SSCS1 and SSCS4 had the highest average ImmuneScore and StromaScore compared to the remaining SSCS subtypes, respectively (Figure S5). Pairwise comparisons of StromaScores among the SSCS subtypes indicated that the differences between SSCS4 and the other four subtypes were all significant (adjusted *p*-value < 0) (Figure S5). Only SSCS1 and SSCS5 have significant differences in their ImmuneScores (adjusted *p*-value < 0). SSCS4 also has the highest average MicroenvironmentScore, which is the summation of the ImmuneScore and StromaScore. All pairwise comparisons of MicroenvironmentScore between SSCS4 or SSCS1 with the remaining subtypes were significant (adjusted *p*-value < 0) (Figure S5). The pairwise comparisons of the ImmuneScore, StromaScore, and MicroenvironmentScore were also evaluated in the CPTAC and GEO cohorts (Figure S5). The SSCS4 stands out to have the highest TME scores, which indicated that the patients classified into the SSCS4 subtype have relatively lower tumor purities.

From the TCGA cohort, we found the abundances of a subset of TME cell types have prognostic power as determined by using the univariate Cox analysis (Wald test *p*-value < 0.05). More specifically, of the 64 cell types investigated, neuron was identified to be significantly associated with poor DFS and OS ((beta coefficients > 0; Wald test *p*-value < 0.05; Table S7). Of note, neuron is one of the main active cell types within the enteric nervous system (ENS), which is interrelated with the immune system [32]. Fibroblast was significantly associated with poor DFS (beta coefficients > 0; Wald test *p*-value < 0.05; Table S7), whereas memory CD4+ T and conventional dendritic cells (cDC) were significantly associated with favorable DFS (beta coefficients < 0; Wald test *p*-value < 0.05; Table S7). Two other cell types, namely naive B and naive CD4+ T cells, were identified to be significantly associated with poor OS (beta coefficients < 0; Wald test *p*-value < 0.05; Table S7).

**Figure 6:**
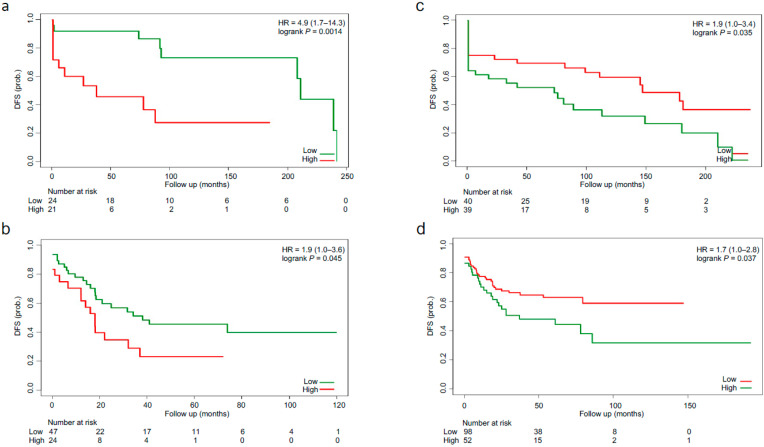
Two Cox models built by using the TME-associated fibroblast and cDC. A Cox model built by using tumor-infiltrating fibroblast in the TCGA SSCS4 patients (*n* = 45), which can be used to classify the SSCS4 patients both from the TCGA (**a**) and GEO (**b**) cohorts into high- and low-risk groups. Another Cox model built by using the TME-associated cDC in the TCGA SSCS3 patients (*n* = 79), which can be used to classify the SSCS3 patients both from the TCGA (**c**) and GEO (**d**) cohorts into high- and low-risk groups.

We next evaluated the six TME cell types (neuron, fibroblast, memory CD4+ T, cDC, naive B and naive CD4+ T cells) prognostic performances within the SSCS subtyping system. For each cell type, Cox regression models were built to stratify the patients within each SSCS subtype, and log-rank tests were utilized to test the survival differences between the high- and low-risk groups, respectively. No cell types can be used to stratify the patients into prognostic groups for OS (Table 2). Two cell types (fibroblast and cDC) were identified to be useful for identifying high- and low-risk groups within the SSCS subtyping system for DFS (log-rank *p* < 0.05; Table 2). More specifically, tumor-infiltrating fibroblast was found to be predictive for poor DFS only within the SSCS4 subtype (Table 2). From the TCGA SSCS4 patients (*n* = 45), we built a Cox model and calculated the median risk score (−0.059). The model was applied to classify the SSCS4 patients both from the TCGA (*n* = 45) and GEO (*n* = 79) cohorts into high-risk (>−0.059) and low-risk (<−0.059) groups. Compared with patients with low-risk scores, patients with high-risk scores had shorter DFS (TCGA: HR 4.9, 95% CI 1.7–14.3, *p* = 0.001; GEO: HR 1.9, 95% CI 1.0–3.6, *p* = 0.045) (Figure 6a,b). cDC, on the contrary, was associated with favorable DFS in the SSCS3 subtype. Cox model was built by using the abundance of the cDC, and patients were classified into high-risk (<0.054) and low-risk (>0.054) groups based on the median risk score (0.054) determined in the SSCS3 subtype from the TCGA cohort (*n* = 79). Patients with low-risk scores had favorable DFS (TCGA: HR 1.9, 95% CI 1.0–3.4, *p* = 0.035; GEO: HR 1.7, 95% CI 1.0–2.8, *p* = 0.037) (Figure 6c,d).

## 4. Discussion

We systematically evaluated the molecular and clinical implications of three previous CRC subtyping systems: TNM stage, MSI and CMS. TNM stage is one of the most important determinants of survival, and it is essential for complementarity with other established systems for better treatment decision-making in CRC. Although MSI-H has been used as a biomarker for immunotherapy and predicting prognosis, MSI-H only accounts for about 15%, and substantial heterogeneity still exists within the remaining 85% non-MSI-H patients. CMS is a well-defined classification system [18], but we and others [19,20,21] have found its applications in advanced-stage CRC cases is limited. Revealing CRC heterogeneity based solely on TNM stage, MSI, or CMS is incomplete. When any of the two systems were considered simultaneously, however, only applicable for a subset of the patients. More specifically, TNM stage + MSI for non-MSI-H cases and TNM stage + CMS for early stage patients. TNM stage provides additional insight into CRC heterogeneity, and remains an influential factor for CRC subtype analysis.

Variations in genomic features and clinical outcomes have been observed in CRC within the same tumor stage. One of the best examples is that De Sousa et al. defined three molecular and clinical distinct subtypes from a cohort of 90 patients with stage II CRC [15]. Previous studies [33,34] have identified several stage-independent variables such as MSI status and tumor grade, which correlated with patients’ clinical outcomes. For example, as mentioned earlier, MSI-H patients, who usually have good prognosis, are more common in early stages than late stages of CRC. Indeed, in our study, we found that a higher percentage of MSI-H patients were distributed into the SSCS1 subtype, which derived from early cases and have favorable clinical outcomes. Moreover, early- and advanced-cases have significant clinical and molecular differences only for non-MSI-H patients. According to several multivariate statistical analyses [33,34], higher tumor grade is a stage-independent prognostic factor in CRC associated with adverse outcomes. In the current study, due to lack of the information, we did not take the tumor grade into consideration when classifying the patients. Future investigations of grade relating to the CRC heterogeneity is needed.

In the current study, we developed a new CRC subtyping system SSCS, which takes the TNM stage as an independent factor. Although the four TNM stages of CRC generally showed significant differences in both DFS and OS (Figure S1), the assignment of tumor stage for each patient is subjective and depends on experience. As there are no molecular level information involved in the four TNM stages, in our study, we did a binary split of the TNM stage into early- and advanced-stages (advanced cases have local or distant metastasis, which is distinguishable from the early cases). By integrating supervised stage information into the unsupervised machine learning frameworks, a total of five CRC subtypes (SSCS1-5) were identified and described. Patients with early-stage CRC were classified into two subtypes (SSCS1-2), which generally have good prognosis. MSI-H patients were basically classified into the SSCS1 subtype. Multiple cell cycle and metabolism-related gene sets were upregulated, and ECM related gene sets were down-regulated in the SSCS2 subtype. Advanced-stage CRC is either metastatic or locally invasive at the time of diagnosis. Three advanced-stage CRC subtypes (SSCS3-5) were identified from the study. Although SSCS3 and SSCS2 subtypes have been enriched with similar functional categories of gene sets, SSCS3 patients have relatively worse survival compared to SSCS2 patients. SSCS4 subtype has low tumor purities and was associated with the worst clinical outcomes. Of note, high frequency of the mesenchymal CMS4 patients were classified into the SSCS4 subtype, indicating the reliability and validity of our results. The SSCS5 subtype has relatively good survival compared to the other two advanced-stage subtypes, and was enriched with amplicon-associated gene sets.

Tumor microenvironment consists of multiple cell types including tumor cells, fibroblasts, lymphocytes, and many other cell types. The non-cancerous cells are important in cancer treatment as well as considered to be independent prognostic factors for clinical outcome prediction. For example, tumor-infiltrating lymphocyte (TIL) therapy is by far the most effective treatment for advanced melanoma [35]. Tumor-infiltrating fibroblasts, or called cancer associated fibroblasts (CAFs), are one of the key components within the TME, which is involved in the formation of the extracellular matrix in tumor tissues. Integrating CAF-targeted agents with existing therapies could provide benefit in treating cancer patients [36]. Moreover, activated tumor-infiltrating fibroblasts correlated with lymph node metastasis, and can be used to predict worse DFS and OS in breast cancer patients [37]. High levels of TILs were associated with improved survival in CRC [38]. In our study, we investigated 64 TME cell types, and determined the relevance of the abundance of two cell types to clinical outcomes within the SSCS subtyping system. More specifically, tumor-infiltrating fibroblast was found to be predictive for poor DFS only within the SSCS4 subtype. cDC, which are antigen-presenting cells that drive T-cell cytokine responses [39], were associated with favorable DFS in the SSCS3 subtype. Further exploration of the subtype specific TME cells has the potential to be novel therapeutic targets for the treatment of CRC.

## 5. Conclusions

In summary, CRC is heterogeneous and should not be treated as a single disease. Histopathological features such as the TNM stage can be useful for unsupervised classification of CRC. Our newly proposed CRC subtyping system SSCS could not only yield better stratification results than the current standards, but also help us gain more insights into CRC heterogeneity and its implications for therapy.

## Figures and Tables

**Table 1:** Patient’s distributions from three previous CRC subtyping systems.

System	Label	Character	Classification	Disadvantage	TCGA	CPTAC	GEO
Stage	Early	Better prognosis	Supervised	No molecular basis	247 (56.7%)	54 (51.4%)	485 (53.1%)
	Advanced	ECM and cell proliferation pathways up			189 (43.3%)	51 (48.6%)	429 (46.9%)
MSI	MSI-H	Immune pathways up	Supervised	High heterogeneity within the non-MSI-H group	70 (16.0%)	24 (22.8%)	0 (0)
	Non-MSI-H	Significant proportion of patients			366 (84.0%)	81 (77.2%)	914 (100.0%)
CMS	CMS1	MSI-H	Unsupervised	Has an unassigned group	59 (13.5%)	14 (13.3%)	44 (4.8%)
	CMS2	Canonical cancer pathways up			150 (34.4%)	33 (31.4%)	369 (40.4%)
	CMS3	Metabolic			50 (11.5%)	16 (15.3%)	105 (11.5%)
	CMS4	EMT			104 (23.9%)	21 (20.0%)	212 (23.2%)
	NOLBL	Unassigned			73 (16.7%)	21 (20.0%)	184 (20.1%)
Stage + MSI	Early_MSI-H	More MSI-H patients	Supervised	Non-distinction between early- and advanced-cases in MSI-H patients	53 (12.1%)	14 (13.3%)	-
	Early_non-MSI-H	Oxidation pathway up			194 (44.5%)	40 (38.1%)	-
	Advanced_MSI-H	Fewer patients			17 (3.9%)	10 (9.5%)	-
	Advanced_non-MSI-H	Worst prognosis			172 (39.5%)	41 (39.1%)	-
Stage + CMS	CMS1-4 (in early-stage)	Distinctive	Supervised	CMS with poor performances in advanced case	214 (59.0%)	47 (56.0%)	405 (55.5%)
	CMS1-4 (in advanced-stage)	Non-distinctive			149 (41.0%)	37 (44.0%)	325 (44.5%)
SSCS	SSCS1	MSI-H	Supervised + unsupervised	No tumor grade information	129 (29.6%)	29 (27.6%)	257 (28.1%)
	SSCS2	ECM pathway down; better prognosis			118 (27.1%)	25 (23.8%)	228 (25.0%)
	SSCS3	Cell cycle pathway up; cDC infiltration			79 (18.1%)	20 (19.1%)	183 (20.0%)
	SSCS4	Fibroblast infiltration; worst prognosis			45 (10.3%)	7 (6.7%)	79 (8.6%)
	SSCS5	Amplicon pathway up			65 (14.9%)	24 (22.8%)	167 (18.3%)

**Table 2:** Log rank *p*-values from survival analysis for the six TME cell types.

	Subtype	Neuron	Fibroblast	Memory CD4+ T	cDC	Naive B	Naive CD4+ T
DFS	SSCS1	0.062	0.500	0.980	0.320	0.680	1
	SSCS2	0.080	1	0.130	0.330	0.130	1
	SSCS3	0.200	1	0.059	0.008 **	0.830	1
	SSCS4	0.290	0.001 **	0.540	0.570	0.870	1
	SSCS5	0.710	1	0.320	0.520	1	0.540
	All	0.064	0.120	0.090	0.008 **	0.140	0.460
OS	SSCS1	0.710	0.190	0.510	0.810	0.750	1
	SSCS2	0.260	1	0.086	0.630	0.390	1
	SSCS3	0.170	1	0.190	0.058	0.450	0.870
	SSCS4	0.270	0.340	0.120	0.560	0.310	0.620
	SSCS5	0.580	0.200	0.058	0.570	1	1
	All	0.100	0.210	0.610	0.510	0.810	0.720

** Significant difference *p* < 0.01.

## Data Availability

Public datasets were downloaded from the TCGAcrcmRNA package (version 1.14.0), LinkedOmics (http://linkedomics.org/cptac-colon/; Accessed on 15 August 2019), and cBioPortal (http://www.cbioportal.org; Accessed on 15 August 2019).

## Supplementary Materials

The following are available online at https://www.mdpi.com/article/10.3390/biomedicines9121815/s1, Figure S1. Kaplan-Meier survival curves for the 4 TNM stages. Figure S2: Multivariate COX PH forest plots for OS, Figure S3: Kaplan–Meier survival curves for the Stage_MSI and Stage_CMS systems, Figure S4: Kaplan–Meier survival curves and multivariate COX PH forest plots for OS, Figure S5: Boxplots of the immune, stroma, and microenvironment scores across the five SSCS subtypes, Table S1: 92 dysregulated gene sets between advanced- and early stage of CRC identified from the three cohorts, Table S2: 537 dysregulated gene sets between MSI-H and non-MSI-H cases identified from the TCGA and CPTAC cohorts, Table S3: 94 dysregulated gene sets between early_MSI-H, early_non-MSI-H, advanced_MSI-H, and advanced_non-MSI-H identified from the TCGA and CPTAC cohorts, Table S4: 89 dysregulated gene sets between CMS 1-4 (in early stage) and CMS 1-4 (in advanced-stage) identified from the three cohorts, Table S5: Selected gene subsets for pre-selection, consensus clustering, and classifier building, Table S6: 103 dysregulated gene sets between the five SSCS subtypes, Table S7: Significant tumor-infiltrating cell types associated with DFS and OS.
