# Supplementary material for: SSCS: A Stage Supervised Subtyping System for Colorectal Cancer
Source: Biomedicines. 2021 Dec 2;9(12):1815. doi: 10.3390/biomedicines9121815 (PMC8698601; doi:10.3390/biomedicines9121815)
Supplement: Supplementary file 1 [file biomedicines-09-01815-s001.zip › biomedicines-1439819 supplementary.pdf]

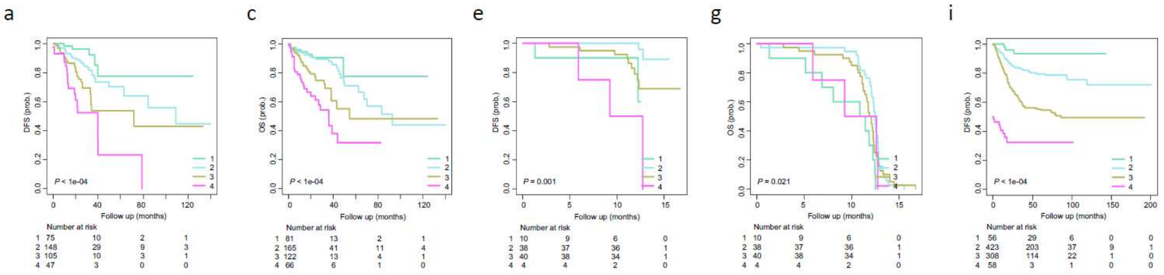

b

	Stage 1	Stage 2	Stage 3
Stage 2	0.071	-	-
Stage 3	0.001	0.022	-
Stage 4	1.1×10^{-6}	5.3×10^{-6}	0.022

d

	Stage 1	Stage 2	Stage 3
Stage 2	0.382	-	-
Stage 3	0.010	0.011	-
Stage 4	1.0×10^{-5}	3.8×10^{-7}	0.019

f

	Stage 1	Stage 2	Stage 3
Stage 2	0.018	-	-
Stage 3	0.787	0.028	-
Stage 4	0.590	3.8×10^{-6}	0.056

h

	Stage 1	Stage 2	Stage 3
Stage 2	0.003	-	-
Stage 3	0.203	0.409	-
Stage 4	0.301	0.301	0.622

j

	Stage 1	Stage 2	Stage 3
Stage 2	0.019	-	-
Stage 3	3.9×10^{-6}	7.6×10^{-12}	-
Stage 4	2.7×10^{-12}	$<2.0 \times 10^{-16}$	1.7×10^{-13}

Figure S1. Kaplan-Meier survival curves for the 4 TNM stages. Kaplan-Meier survival curves comparing DFS of the four stages (1: stage I; 2: stage II; 3: stage III; 4: stage IV) in the TCGA (a), CPTAC (e), and GEO (i) cohorts. The indicated p-values were calculated with the log-rank tests. Kaplan-Meier survival curves comparing OS of the four stages (1: stage I; 2: stage II; 3: stage III; 4: stage IV) in the TCGA (c) and CPTAC (g) cohorts. The indicated p-values were calculated with the log-rank tests. Summary tables (b, d, f, h, and j) of the pairwise comparisons of survival curves between the four stages in different cohorts. Survival differences were tested using the log-rank tests, with FDR adjusted p-values less than 0.05 were considered statistically significant and displayed in red color.

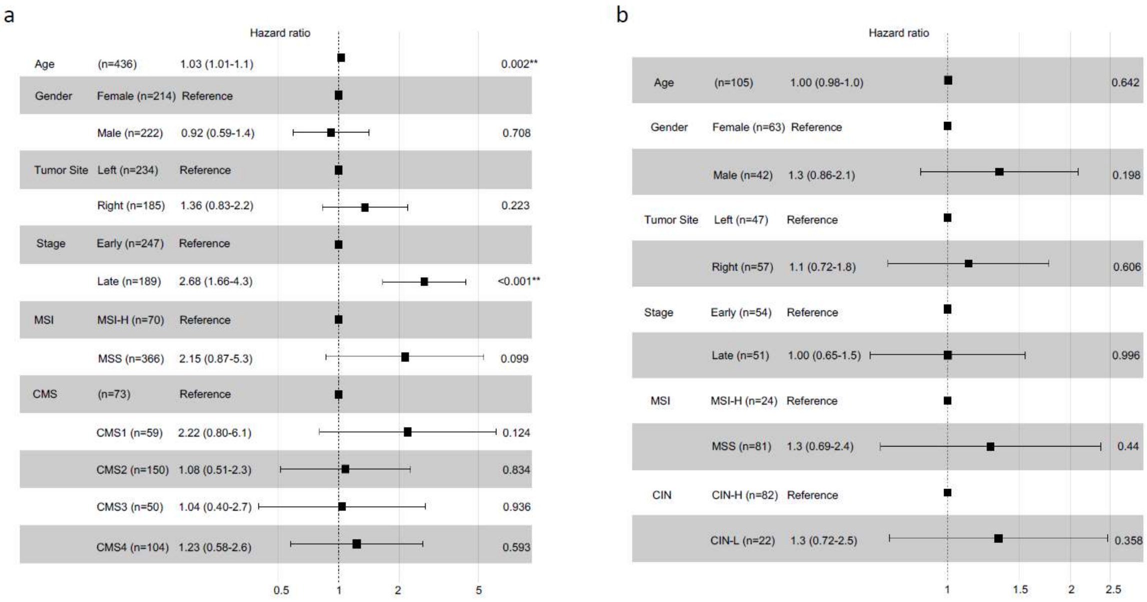

Figure S2. Multivariate COX PH forest plots for OS. Forest plots of Cox PH regression models illustrated the HRs, 95% CIs and log-rank p-values for different subtyping systems (stage, MSI, CMS, etc.) and confounder factors (age, gender, etc.) for OS in the TCGA (a) and CPTAC (b) cohorts. Log-rank p-value significance levels were given by stars: * < 0.05, and ** < 0.01.

a

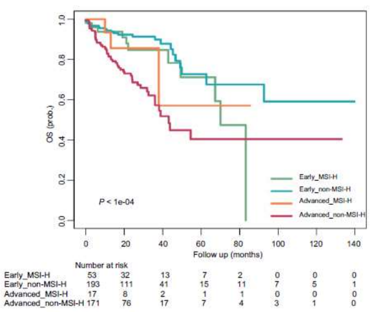

b

	Early_MSI-H	Early_non-MSI-H	Advanced_MSI-H
Early_non-MSI-H	0.33	-	-
Advanced_MSI-H	0.92	0.40	-
Advanced_non-MSI-H	0.11	9.5×10^{-7}	0.33

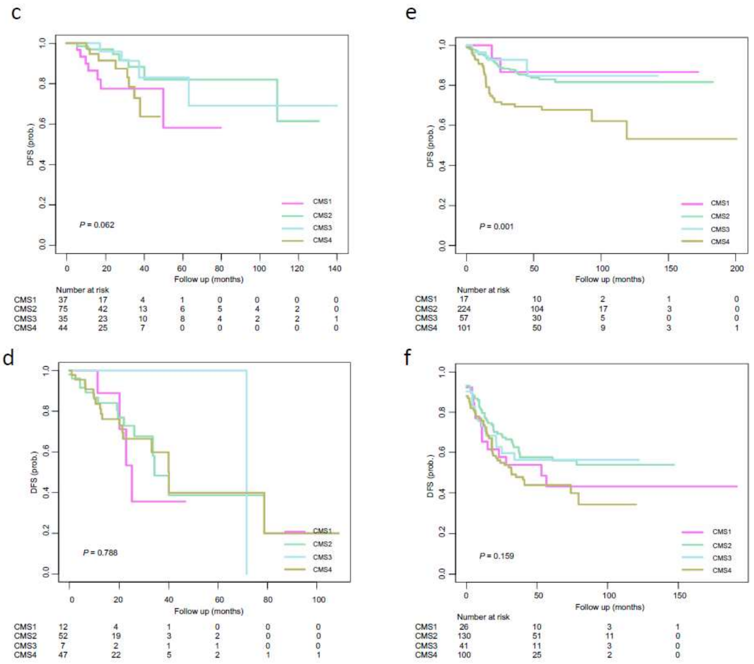

Figure S3. Kaplan-Meier survival curves for the Stage_MSI and Stage_CMS systems. (a). Kaplan-Meier survival curve comparing OS of the four groups (Early_MSI-H, Early_non-MSI-H, Advanced_MSI-H, and Advanced_non-MSI-H) in the TCGA cohort. The indicated p-value was calculated with the log-rank test. (b). Summary table of the pairwise comparisons of survival curves between the four groups. Survival difference was tested using the log-rank test, with FDR adjusted p-values less than 0.05 were considered statistically significant and displayed in red color. Kaplan-Meier survival curves comparing DFS of the four CMS subtypes (CMS1-4) in the TCGA_early (c), TCGA_advanced (d), GEO_early (e), and GEO_advanced (f) cases. The indicated p-values were calculated with the log-rank tests.

a

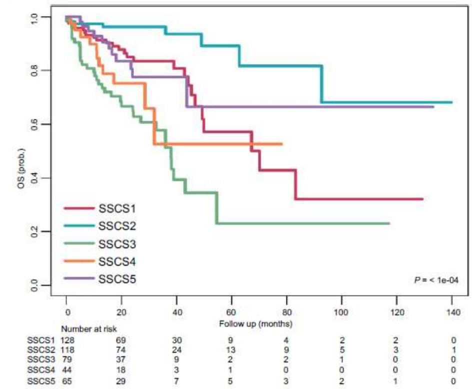

b

	SSCS1	SSCS2	SSCS3	SSCS4
SSCS2	0.002	-	-	-
SSCS3	0.001	6.1×10^{-10}	-	-
SSCS4	0.166	8.5×10^{-5}	0.289	-
SSCS5	0.871	0.013	0.009	0.255

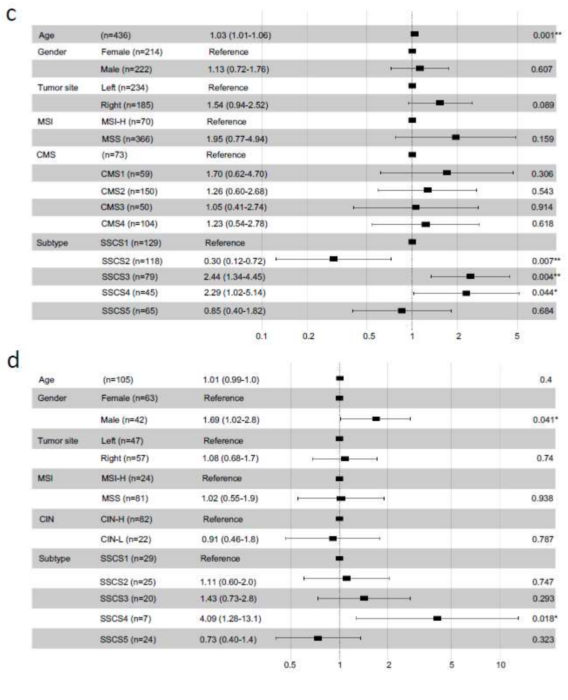

Figure S4. Kaplan-Meier survival curves and multivariate COX PH forest plots for OS. (a). Kaplan-Meier survival curve comparing OS of the five SSCS subtypes in the TCGA cohort. The indicated p-value was calculated with the log-rank test. (b). Summary table of the pairwise comparisons of survival curves between the five SSCS subtypes in the TCGA cohort. Survival differences were tested using the log-rank tests, with FDR adjusted p-values less than 0.05 were considered statistically

significant and displayed in red colors. Forest plots of Cox PH regression models illustrated the HRs, 95% CIs and log-rank p-values for different subtyping systems (SSCS, MSI, CMS, etc.) and confounder factors (age, gender, etc.) for OS in the TCGA (c) and CPTAC (d) cohorts. Log-rank p-value significance levels were given by stars: * < 0.05, and ** < 0.01.

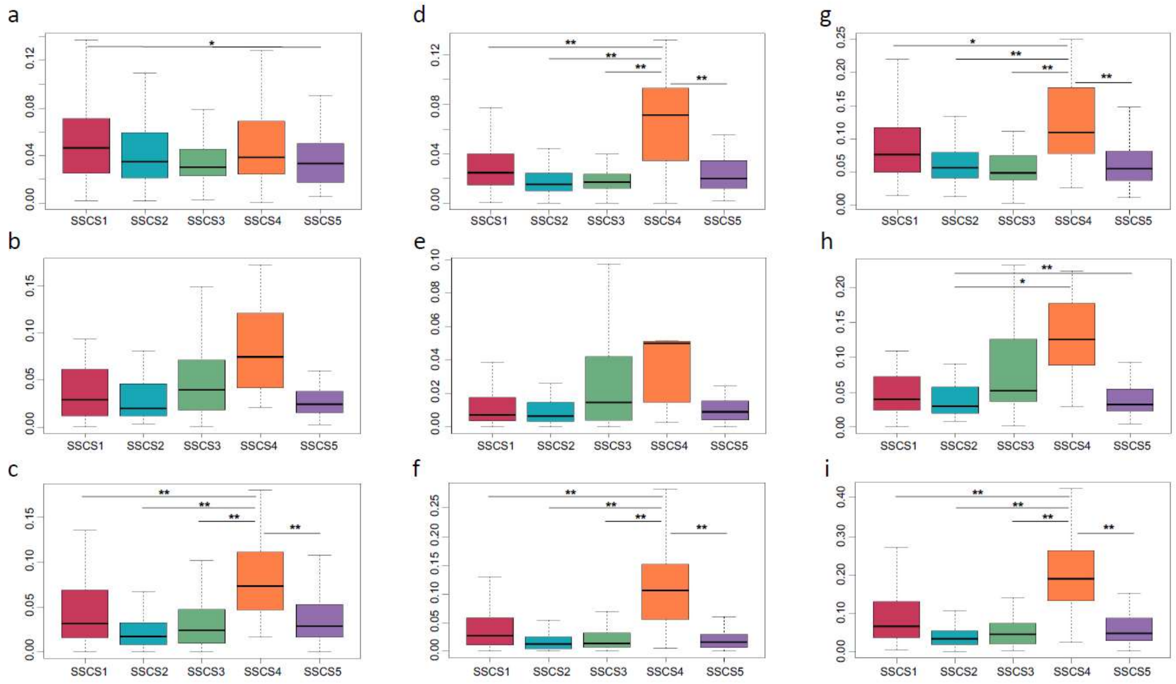

Figure S5. Boxplots of the immune, stroma, and microenvironment scores across the five SSCS subtypes. Boxplots of the immune (a-c), stroma (d-f), and microenvironment (g-i) scores across the five SSCS subtypes. Patient cohorts include TCGA (a, d, g), CPTAC (b, e, h), and GEO (c, f, i). Pairwise Wilcoxon rank sum tests with FDR adjusted p-value < 0.05 were considered statistically significant, and marked with a star. Two stars represent FDR adjusted p-value < 0.01.
