# Supplementary material for: SSCS: A Stage Supervised Subtyping System for Colorectal Cancer
Source: Biomedicines. 2021 Dec 2;9(12):1815. doi: 10.3390/biomedicines9121815 (PMC8698601; doi:10.3390/biomedicines9121815)
Supplement: Supplementary file 1 [file biomedicines-09-01815-s001.zip › Figure S1_final.pdf]

a

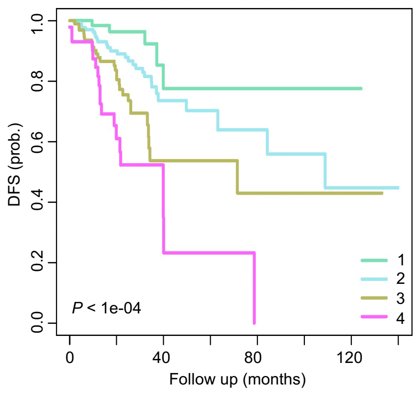

Number at risk

	0	40	80	120
1	75	10	2	1
2	148	29	9	3
3	105	10	3	1
4	47	3	0	0

c

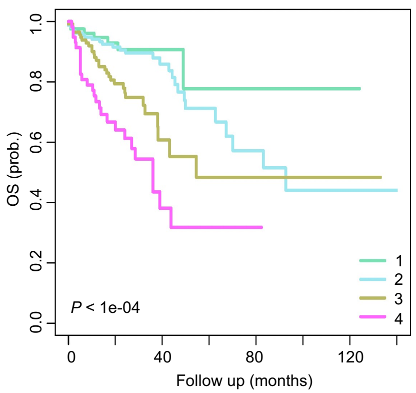

Number at risk

	0	40	80	120
1	81	13	2	1
2	165	41	11	4
3	122	13	4	1
4	66	6	1	0

e

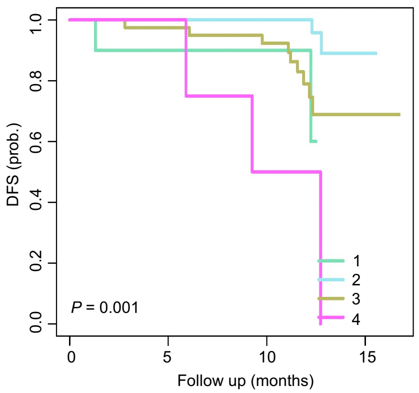

Number at risk

	0	5	10	15
1	10	9	6	0
2	38	37	36	1
3	40	38	34	1
4	4	4	2	0

g

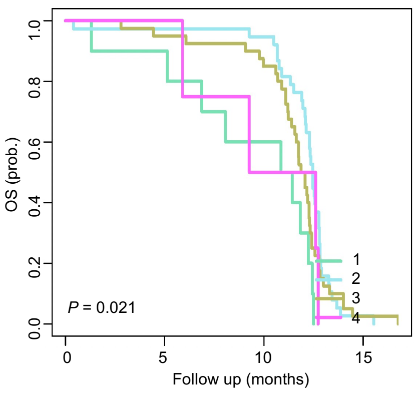

Number at risk

	0	5	10	15
1	10	9	6	0
2	38	37	36	1
3	40	38	34	1
4	4	4	2	0

i

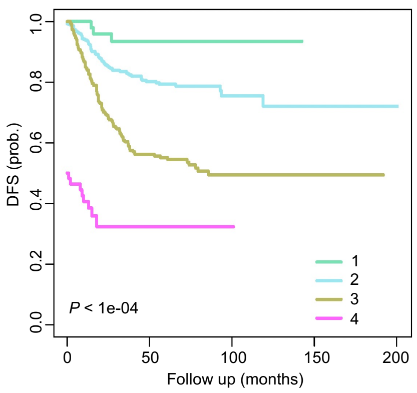

Number at risk

	0	50	100	150	200
1	56	29	6	0	0
2	423	203	37	9	1
3	308	114	22	1	0
4	58	3	1	0	0

b

	Stage 1	Stage 2	Stage 3
Stage 2	0.071	-	-
Stage 3	0.001	0.022	-
Stage 4	1.1×10^{-6}	5.3×10^{-6}	0.022

d

	Stage 1	Stage 2	Stage 3
Stage 2	0.382	-	-
Stage 3	0.010	0.011	-
Stage 4	1.0×10^{-5}	3.8×10^{-7}	0.019

f

	Stage 1	Stage 2	Stage 3
Stage 2	0.018	-	-
Stage 3	0.787	0.028	-
Stage 4	0.590	3.8×10^{-6}	0.056

h

	Stage 1	Stage 2	Stage 3
Stage 2	0.003	-	-
Stage 3	0.203	0.409	-
Stage 4	0.301	0.301	0.622

j

	Stage 1	Stage 2	Stage 3
Stage 2	0.019	-	-
Stage 3	3.9×10^{-6}	7.6×10^{-12}	-
Stage 4	2.7×10^{-12}	$<2.0 \times 10^{-16}$	1.7×10^{-13}
