# Supplementary material for: SSCS: A Stage Supervised Subtyping System for Colorectal Cancer
Source: Biomedicines. 2021 Dec 2;9(12):1815. doi: 10.3390/biomedicines9121815 (PMC8698601; doi:10.3390/biomedicines9121815)

a

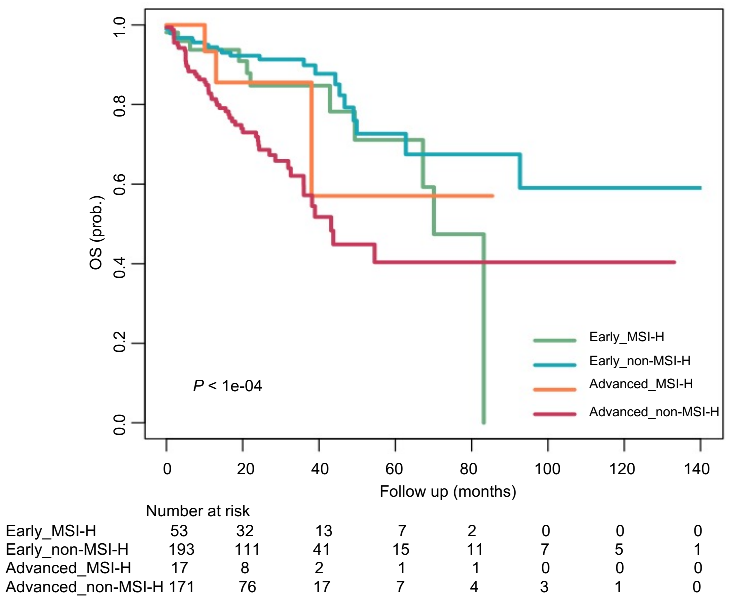

b

	Early_MSI-H	Early_non-MSI-H	Advanced_MSI-H
Early_non-MSI-H	0.33	-	-
Advanced_MSI-H	0.92	0.40	-
Advanced_non-MSI-H	0.11	9.5×10^{-7}	0.33

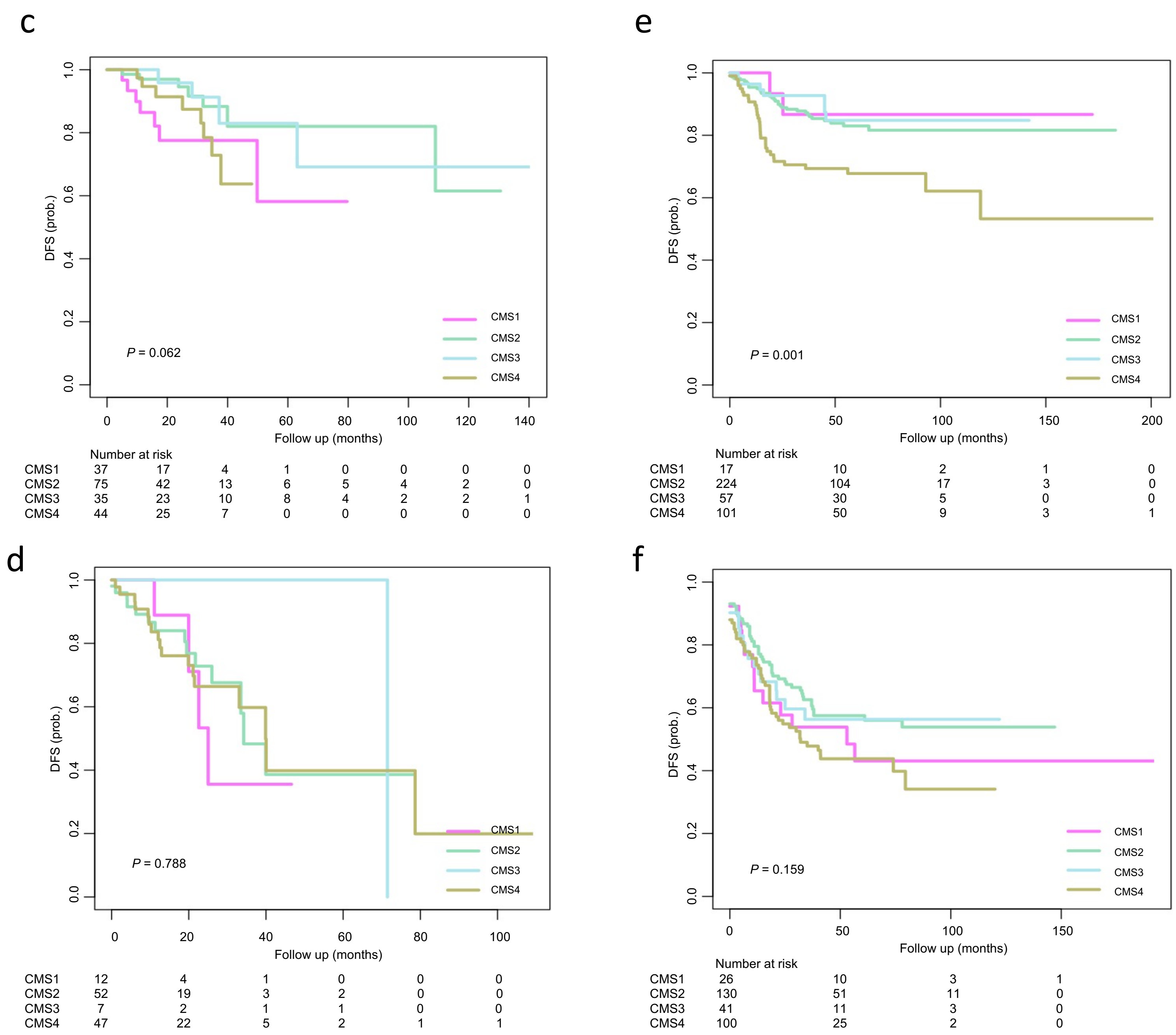

Supplement: Supplementary file 1 [file biomedicines-09-01815-s001.zip › Figure S3_final.pdf]
