# Supplementary figures and images for: SSCS: A Stage Supervised Subtyping System for Colorectal Cancer
Source: Biomedicines. 2021 Dec 2;9(12):1815. doi: 10.3390/biomedicines9121815 (PMC8698601; doi:10.3390/biomedicines9121815)

a

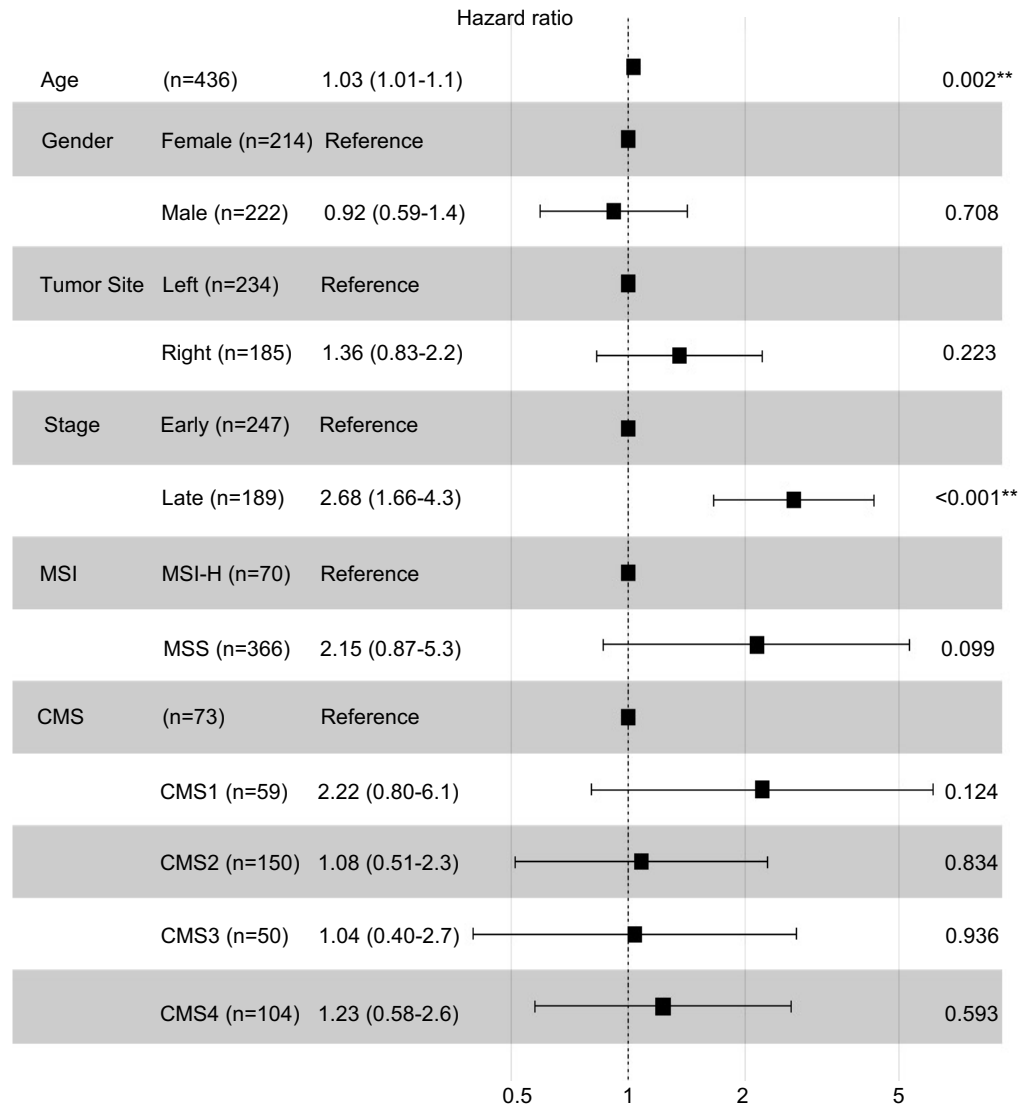

b

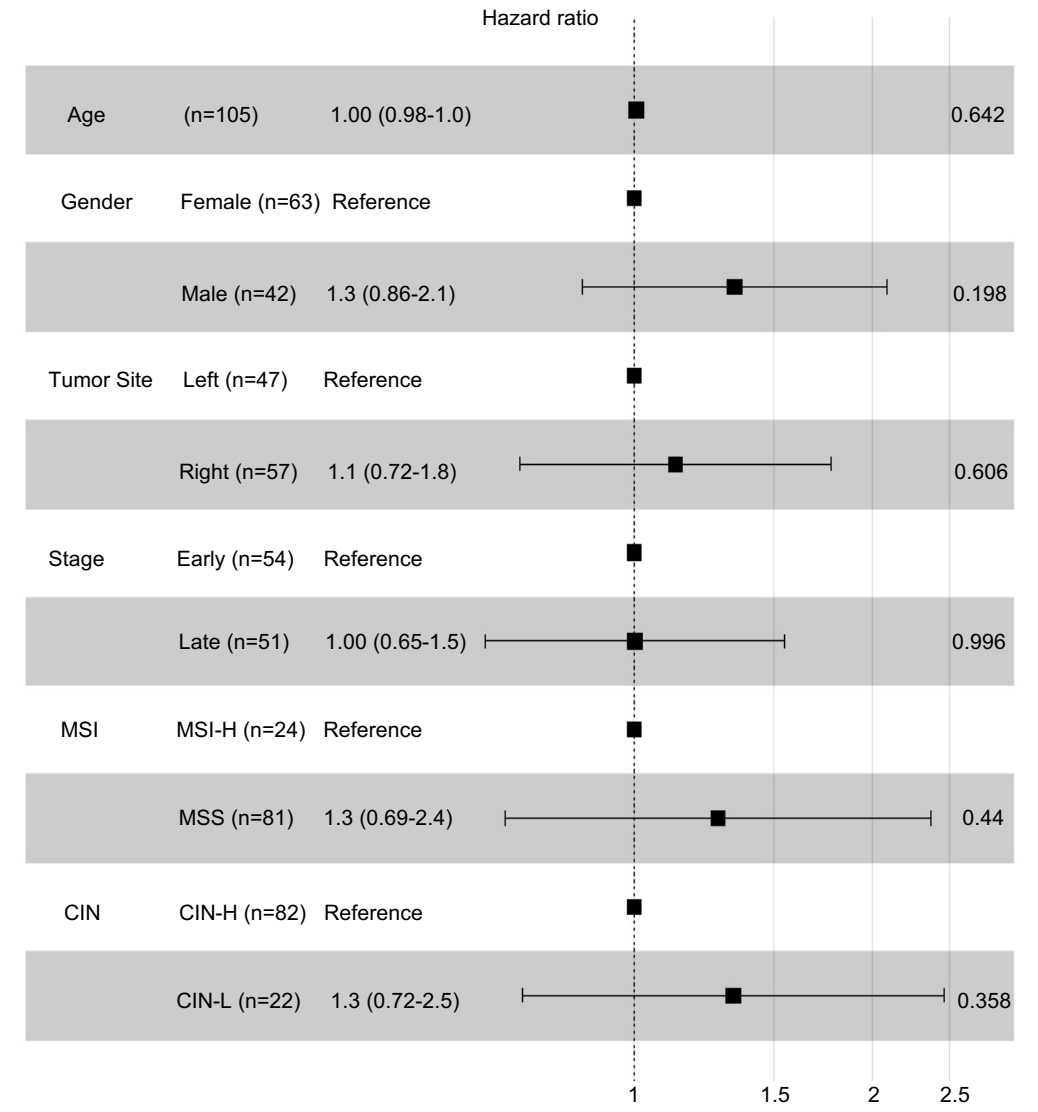

Supplement: Supplementary file 1 [file biomedicines-09-01815-s001.zip › Figure S2_final.pdf]

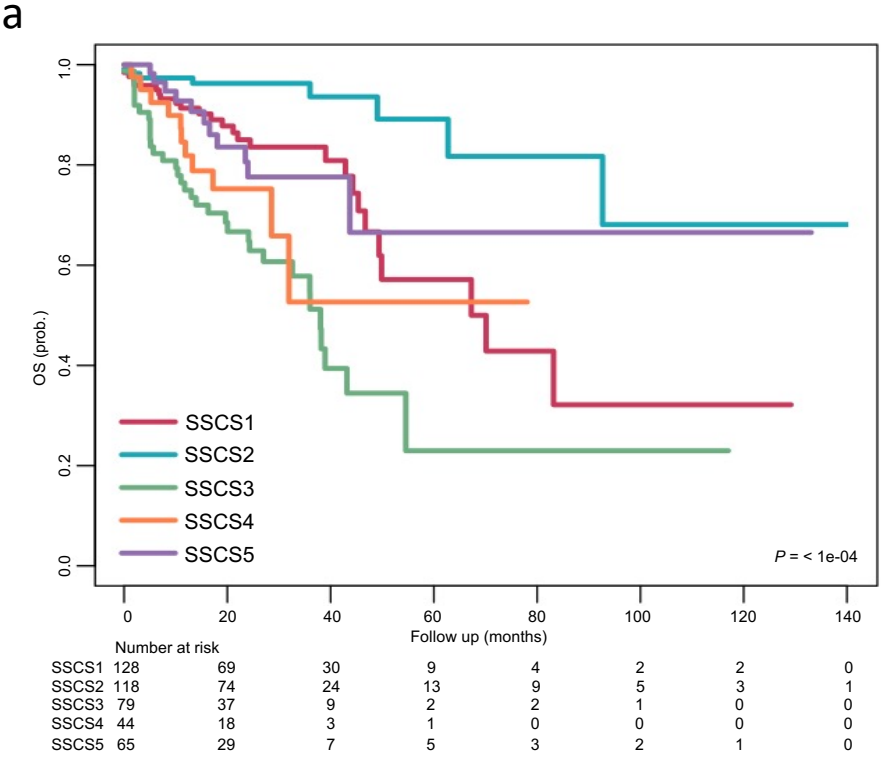

**b**

|       | SSCS1 | SSCS2                 | SSCS3 | SSCS4 |
|-------|-------|-----------------------|-------|-------|
| SSCS2 | 0.002 | -                     | -     | -     |
| SSCS3 | 0.001 | $6.1 \times 10^{-10}$ | -     | -     |
| SSCS4 | 0.166 | $8.5 \times 10^{-5}$  | 0.289 | -     |
| SSCS5 | 0.871 | 0.013                 | 0.009 | 0.255 |

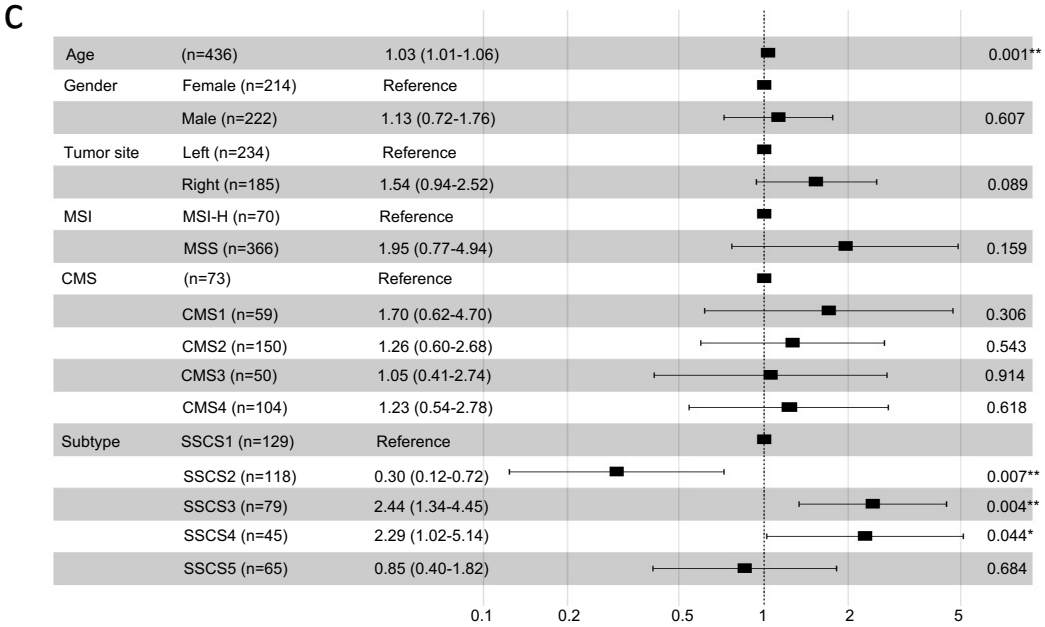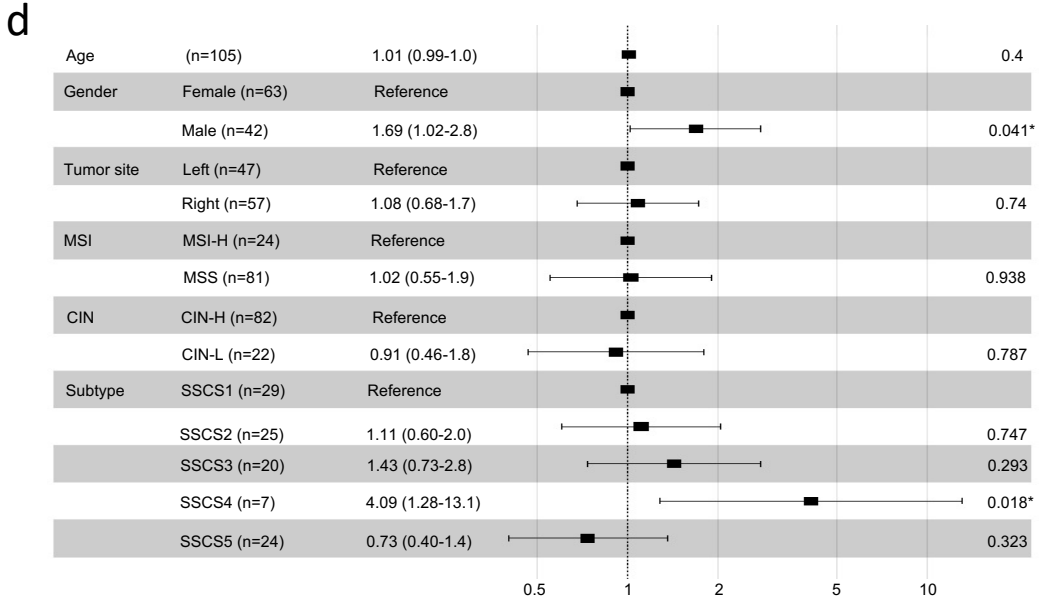

Supplement: Supplementary file 1 [file biomedicines-09-01815-s001.zip › Figure S4_final.pdf]

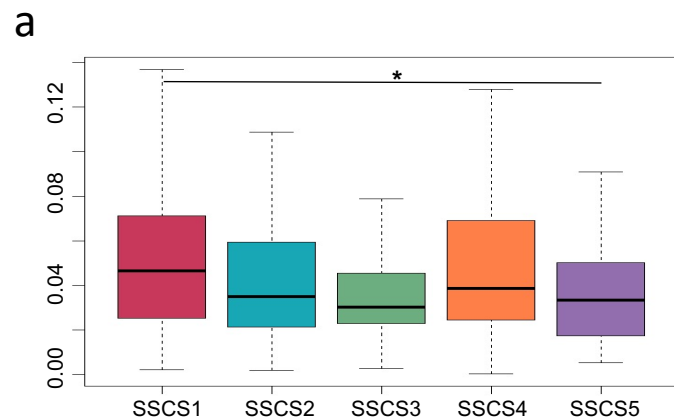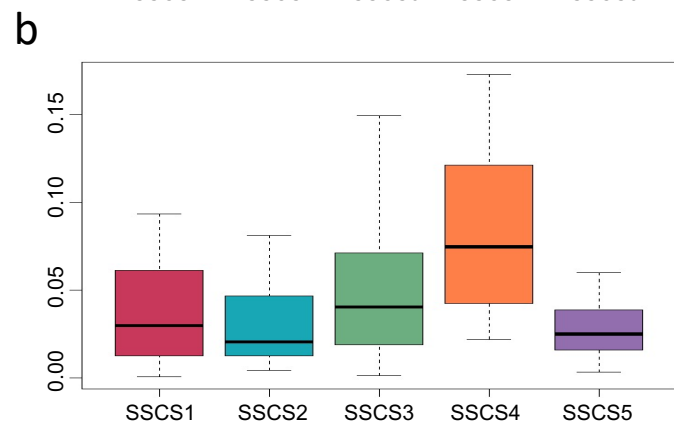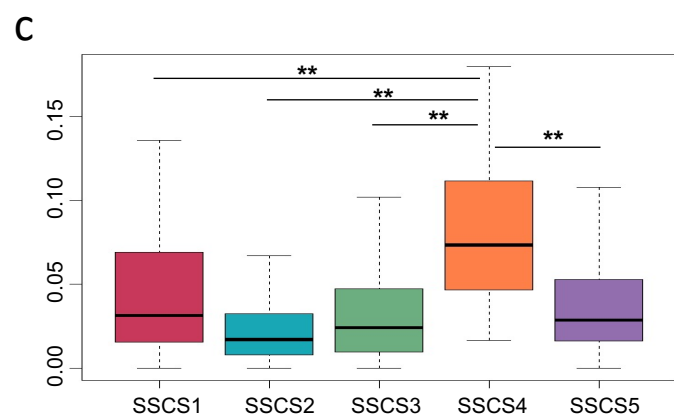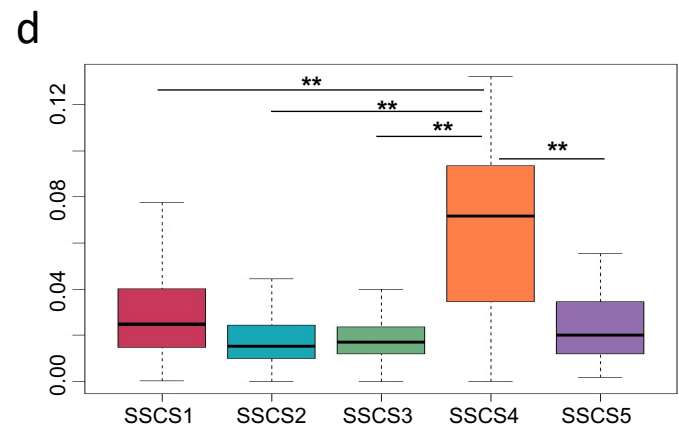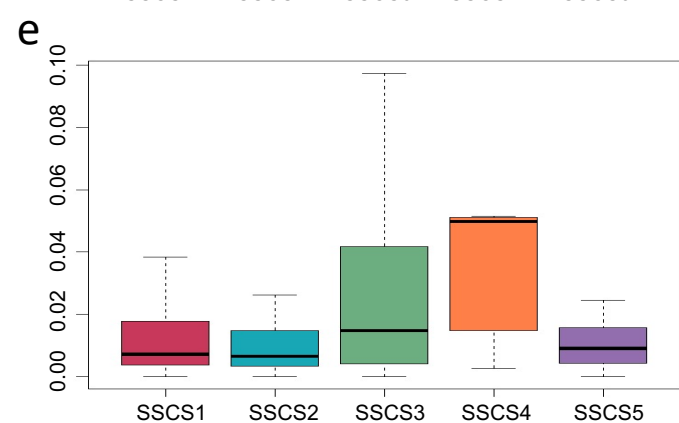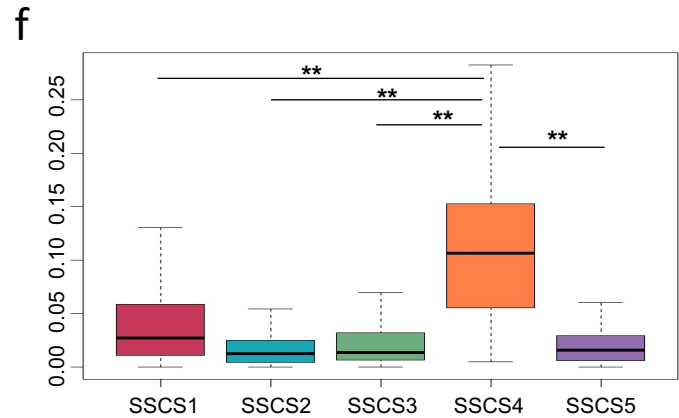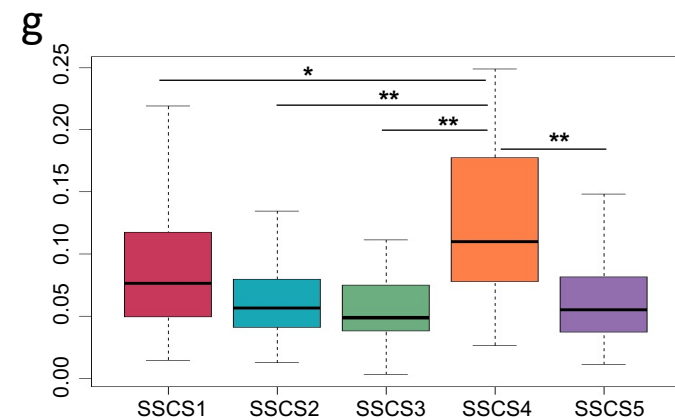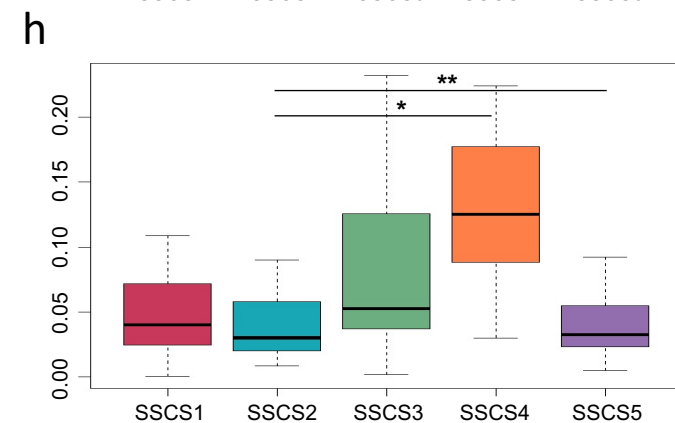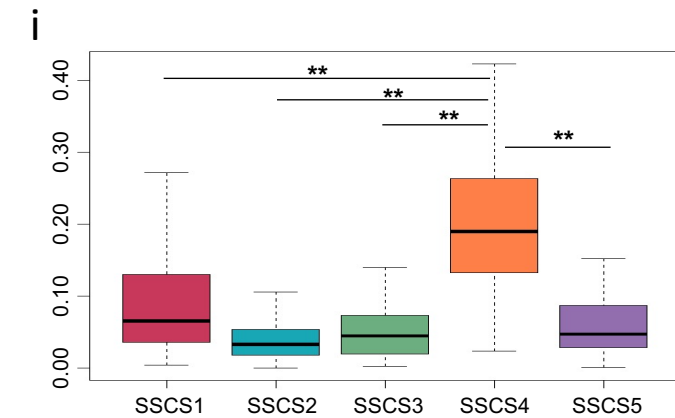

Supplement: Supplementary file 1 [file biomedicines-09-01815-s001.zip › Figure S5_final.pdf]
